# Supplementary material for: Proactive Decision Support for Glaucoma Treatment: Predicting Surgical Interventions with Clinically Available Data
Source: Bioengineering (Basel). 2024 Jan 30;11(2):140. doi: 10.3390/bioengineering11020140 (PMC10886033; doi:10.3390/bioengineering11020140)
Supplement: Supplementary file 1 [file bioengineering-11-00140-s001.zip › Table S1.pdf]

**Table S1:** Summary of all model input features and output in predicting surgical intervention.		
**Feature Type**	**Name**	**Description**
Demographics	Age	Patient age, years
	Sex	Patient sex
	Self-reported Race	Patient race based on self-report
	Self-reported Ethnicity	Patient ethnicity based on self-report
VF	Mean Deviation (MD)	24-2 mean deviation, dB
	Pattern Standard Deviation (PSD)	24-2 pattern standard deviation, dB
	Visual Field Index (VFI)	Visual field index
	PD points	Pattern deviation of individual 24-2 test points
OCT	Mean RNFL Thickness	Mean global retinal nerve fiber layer (RNFL) thickness from optic nerve head (ONH) circle scan. Computed using Spectralis built-in software.
	Sectoral RNFL Thickness	Mean sectoral RNFL thickness computed from ONH circle scans. Sectors: temporal, semporal-superior, temporal-inferior, nasal, nasal-superior, nasal-inferior
Ophthalmic Measurements	Spherical Equivalent (SE)	Spherical equivalent (sphere + ½ cylinder), diopters
	Axial Length (AL)	Axial length
	Central Corneal Thickness (CCT)	Central corneal thickness, mm
	Intraocular Pressure (IOP)	Undilated intraocular pressure, mmHg
Systemic Conditions	-	Self-reported presence of systemic conditions including: myocardial infarction, peripheral vascular disease, peptic ulcer disease, HIV, dementia, congestive heart failure, stroke, pulmonary disease, liver disease, diabetes mellitus, renal disease, cancer, metastatic
Medications	-	Self-reported medications based on anatomical-therapeutic-chemical (ATC) drug classifications from the WHO, retrieved via RxNav. Classes include: glaucoma meds, diabetic meds, hypertension meds, anti-inflammatory meds, alpha adrenoreceptor antagonists, glucocorticoids, lipid modifying agents, dermatologicals, opioids, agents for local oral treatment, carbonic anhydrase inhibitors, propionic acid derivatives, beta blockers, other cardiac preparations, prostaglandin analogues, sympathomimetics, insulins, corticosteroids
Model Output	-	Estimate of probability of surgical intervention. For this analysis, glaucoma surgeries included incisional, laser, or minimally invasive glaucoma surgery (MIGS) procedures.
