# Supplementary material for: Proactive Decision Support for Glaucoma Treatment: Predicting Surgical Interventions with Clinically Available Data
Source: Bioengineering (Basel). 2024 Jan 30;11(2):140. doi: 10.3390/bioengineering11020140 (PMC10886033; doi:10.3390/bioengineering11020140)
Supplement: Supplementary file 1 [file bioengineering-11-00140-s001.zip › Table S2.pdf]

**Table S2:** Performance of all models in predicting surgical interventions at time horizons up to 3 years.					
**Model Type**	**Time Horizon**	**AUC**	**Precision**	**Sensitivity/Recall**	**Specificity**
GBM	3 months	0.94 (0.91 - 0.97)	0.79 (0.71 - 0.87)	0.90 (0.84 - 0.95)	0.84 (0.77 - 0.89)
	1 year	0.95 (0.92 - 0.97)	0.81 (0.72 - 0.89)	0.77 (0.68 - 0.85)	0.88 (0.83 - 0.94)
	2 years	0.95 (0.93 - 0.98)	0.84 (0.76 - 0.91)	0.87 (0.79 - 0.93)	0.90 (0.84 - 0.94)
	3 years	0.95 (0.93 - 0.98)	0.93 (0.85 - 0.98)	0.71 (0.62 - 0.81)	0.97 (0.93 - 0.99)
Random Forest	3 months	0.95 (0.92 - 0.97)	0.84 (0.76 - 0.90)	0.86 (0.78 - 0.93)	0.88 (0.83 - 0.93)
	1 year	0.93 (0.90 - 0.96)	0.81 (0.72 - 0.89)	0.74 (0.65 - 0.83)	0.88 (0.83 - 0.93)
	2 years	0.93 (0.90 - 0.97)	0.90 (0.82 - 0.96)	0.77 (0.67 - 0.85)	0.94 (0.90 - 0.98)
	3 years	0.95 (0.93 - 0.98)	0.93 (0.85 - 0.98)	0.69 (0.60 - 0.79)	0.97 (0.93 - 0.99)
Logistic Regression	3 months	0.94 (0.91 - 0.97)	0.79 (0.71 - 0.87)	0.89 (0.82 - 0.95)	0.84 (0.78 - 0.90)
	1 year	0.90 (0.87 - 0.94)	0.77 (0.68 - 0.85)	0.81 (0.72 - 0 88)	0.84 (0.78 - 0.90)
	2 years	0.86 (0.82 - 0.91)	0.71 (0.61 - 0.80)	0.68 (0.58 - 0.77)	0.83 (0.76 - 0.89)
	3 years	0.83 (0.78 - 0.88)	0.64 (0.53 - 0.73)	0.68 (0.58 - 0.77)	0.77 (0.69 - 0.83)
XGBoost	3 months	0.94 (0.91 - 0.97)	0.82 (0.73 - 0.89)	0.80 (0.71 - 0.88)	0.88 (0.82 - 0.93)
	1 year	0.93 (0.90 - 0.96)	0.82 (0.74 - 0.90)	0.80 (0.71 - 0.88)	0.88 (0.83 - 0.94)
	2 years	0.96 (0.93 - 0.98)	0.92 (0.85 - 0.98)	0.67 (0.56 - 0.76)	0.97 (0.93 - 0.99)
	3 years	0.94 (0.91 - 0.97)	0.90 (0.82 - 0.96)	0.78 (0.69 - 0.86)	0.94 (0.90 - 0.98)
DNN	3 months	0.90 (0.85 - 0.94)	0.61 (0.53 - 0.70)	0.89 (0.82 - 0.95)	0.61 (0.52 - 0.69)
	1 year	0.89 (0.86 - 0.93)	0.77 (0.67 - 0.86)	0.74 (0.65 - 0.83)	0.86 (0.80 - 0.91)
	2 years	0.90 (0.87 - 0.94)	0.81 (0.72 - 0.89)	0.71 (0.62 - 0.80)	0.90 (0.84 - 0.94)
	3 years	0.90 (0.86 - 0.94)	0.79 (0.70 - 0.87)	0.79 (0.70 - 0.87)	0.87 (0.81 - 0.92)
GBM: gradient boosting machine, XGBoost: extreme gradient boosting, DNN: deep neural network					
